# Longitudinal Assessment of the Quality of Life in Oral Squamous Cell Carcinoma Patients

**DOI:** 10.7759/cureus.60596

**Published:** 2024-05-19

**Authors:** Priyadharshini G, Karthikeyan Ramalingam, Pratibha Ramani, Murugesan Krishnan

**Affiliations:** 1 Oral Pathology and Microbiology, Saveetha Dental College and Hospitals, Saveetha Institute of Medical and Technical Sciences, Saveetha University, Chennai, IND; 2 Oral and Maxillofacial Surgery, Saveetha Dental College and Hospitals, Saveetha Institute of Medical and Technical Sciences, Saveetha University, Chennai, IND

**Keywords:** oral & maxillofacial pathology, longitudinal study, long term followup, european organization for research and treatment of cancer quality of life questionnaire (eortc qlq-c30), oral and maxillofacial surgery, oral squamous cell carcinoma, oral cancer, qol: quality of life, health related-quality of life, quality of life (qol)

## Abstract

Background

Studies evaluating the quality of life (QoL) among oral cancer patients in the Indian population are scarce. Regular follow-ups and QoL assessment in oral squamous cell carcinoma (OSCC) patients can aid in comprehensive support strategies to improve their QoL outcomes.

Aim and objectives

This study aimed to assess the QoL of oral cancer patients and correlate the QoL with demographic and treatment parameters.

Materials and methods

The study included oral cancer patients who had previously reported to the Department of Oral and Maxillofacial Surgery. QoL assessment was done using the EORTC QLQ-C30 and QLQ-HN43 questionnaires before and after treatment. The clinico-demographic details, treatment data, follow-up data, and recorded mean QoL were procured from the patient records in Dental Information Archival Software. Assessment of QoL was done before treatment and at intervals of one month, three months, six months, 12 months, 24 months, and 36 months postoperatively after treatment. Statistical analysis was performed using IBM SPSS Statistics for Windows, Version 23 (released 2015; IBM Corp., Armonk, New York, United States). A repeated measures analysis of variance (ANOVA) was utilized for comparing the average QoL scores and frequency of follow-ups across various intervals. Chi-square tests assessed differences in mean QoL among genders, across different sites, and between primary closure and graft placement. The significance was set at a p-value of less than 0.05.

Results

A total of 90 OSCC patients had reported to the department. A preoperative assessment of QoL was done for 90 (100%) patients. Out of these patients, surgery has been performed on 41 (45%). Twenty-five out of 41 (60%) patients had responded to regular follow-up, and QoL was assessed for these patients. After the immediate postoperative phase, only 12 (48%) had reported after three months. Only six (24%) had a 12-month follow-up, five (20%) had a two-year follow-up, and one (4%) had a three-year follow-up. There was a constant decrease in the number of follow-ups after the treatment of OSCC (p=0.00). Prior to treatment, the mean QoL index was 4.64. Females had a slightly higher preoperative QoL of 4.76 compared to males, with a score of 4.67 (p=0.157). Immediately after the treatment of OSCC, a decline in QoL scores was noted, with a mean score of 4.25 (p=0.32). Patients who underwent primary closure after excision had a mean post-op QoL score of 4.9, while patients who underwent graft placement had a mean score of 4.6 (p=0.157).

Conclusion

This study highlights the enduring impact of oral cancer on a patient’s quality of life and emphasizes the need for ongoing research to explore specific interventions that can contribute to sustained improvement in QoL. It emphasizes personalized, holistic care approaches for such patients.

## Introduction

One of the most common cancers of the head and neck is oral squamous cell carcinoma (OSCC), which has high incidence rates and increased mortality in different nations. It also has complicated social and economic effects on those who survive this extremely incapacitating disease [1]. It has been reported that about 25-50% of individuals with advanced OSCC experience locoregional recurrence over a five-year follow-up [2]. Around 3-5% of patients each year present with second primary malignancies [3]. Treatment options for malignancies of the oral cavity are numerous and varied. The most often administered treatments are radiotherapy, chemotherapy, and surgery, either separately or in combination [4].

OSCC management often has serious side effects on patients. Mucositis (stomatitis), xerostomia (dry mouth), bacterial, fungal, or viral infections, dental caries, taste loss, osteoradionecrosis, nutritional impairment, anorexia, and malaise are among the important side effects of radiotherapy [5]. Anatomical changes caused by head and neck cancer treatment and surgery can result in significant oral dysfunction, including trouble speaking, eating, and swallowing. Additionally, these treatments may lower their quality of life (QoL) [6].

The main issue in cancer treatment is striking a balance between cure and survival while restoring function, appearance, and QoL. A patient's self-perception about their medical state is one aspect of the multifaceted idea of QoL. The World Health Organization (WHO) contends that a person's physical, mental, and social well-being are related to prominent environmental elements and impact their quality of life [7]. It has been shown that baseline QoL of patients with head and neck cancer and comorbidity influenced post-treatment quality of life more than the treatment modality [8].

It is currently difficult to quantify the effectiveness of treatment regimens for different head and neck malignancies. Uncertainty remains regarding the QoL metrics and the impact of oral cancer treatment on patients' quality of life. QoL assessments of patients with head and neck cancer could be used for treatment planning and add more extensive clinical, social, and rehabilitative support measures [9]. This allows a better understanding of how the condition affects the patient’s daily routine. 

Very few studies have assessed the QoL among OSCC patients in India [7]. Therefore, we performed a preliminary evaluation of QOL using QLQ-C30 and EORTC QLQ-HN43 among OSCC patients and clinical correlation [10]. This questionnaire, recognized for its reliability and validity, facilitated a comprehensive evaluation of the diverse aspects of QoL. Thus, this study aimed to record the QoL of OSCC patients at various intervals over three years and assess the impact of treatment, the subsequent postoperative recovery, and QoL.

## Materials and methods

This study assessed the evolution of QoL in OSCC patients based on their three-year follow-up pattern. The study was performed at Saveetha Dental College, Chennai, India. Ethical clearance was obtained from the institutional review board (IHEC/SDC/PhD/OPATH-1954/19/TH-001). Informed consent was also obtained from the participants before the beginning of the study, and unwilling participants were excluded. The study participants included only histopathologically confirmed OSCC patients. Patients with any other head and neck pathology, trauma, or infections were excluded. 

The study employed EORTC QLQ-C30 and QLQ-HN43 questionnaires as a standardized Quality of Life Index questionnaire, encompassing physical, psychological, social, and environmental domains [10]. A preoperative assessment of QOL was conducted before the initiation of the treatment, and immediate postoperative QOL was also assessed. The continued assessment was done at one month, three months, six months, 12 months, 24 months, and 36 months postoperatively.

Our institute's indigenous software, Dental Information Archival Software (DIAS), recorded the QoL scores of the patients. A score of 1 indicates the worst QOL, while a score of 7 indicates the best QOL among the patients. OSCC management data and clinical details were retrieved from DIAS, and the results were tabulated. Details regarding the gender, site of involvement, nature of surgical intervention, and survival status were assessed.

Gender criteria were male, female, and transgender. Clinical details were used to identify the site of involvement, including the buccal mucosa, alveolar mucosa, floor of the mouth, tongue, palate, and retromolar trigone. The mode of surgical excision management was noted as primary closure and wound closure with graft placement. Survival status was also assessed. If the patient had succumbed to the disease, the site of involvement, invasion of loco-regional tissues like skin involvement, and tumor staging at the time of diagnosis were evaluated. Relevant clinical pictures were also retrieved from DIAS.

The mean QoL scores were calculated for all the study patients. Statistical analyses were performed using the IBM SPSS Statistics for Windows, Version 23 (released 2015; IBM Corp., Armonk, New York, United States). A repeated measures analysis of variance (ANOVA) was employed to compare the mean QoL scores and the number of follow-ups at different intervals. A chi-square test was done to compare the mean Qol between males and females, between different involved sites, and between primary closure and placement of the graft. Significant values were set at p less than 0.05.

## Results

**Figure 1:**
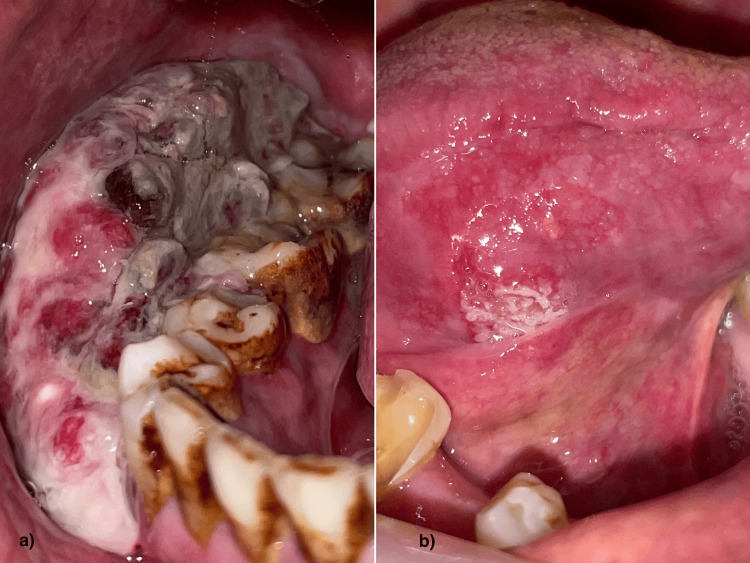
Clinical pictures a: intra-oral picture showing proliferative growth in the vestibule; b: clinical picture showing ulcero-proliferative growth on the ventral surface of the tongue.

This study included only patients with a histopathological diagnosis of oral squamous cell carcinoma. We had an equal distribution of males and females in the study group. No transgender patients were noted. In the follow-up period, we also observed recurrence after surgery among the included patients (Figure 1).

**Figure 2:**
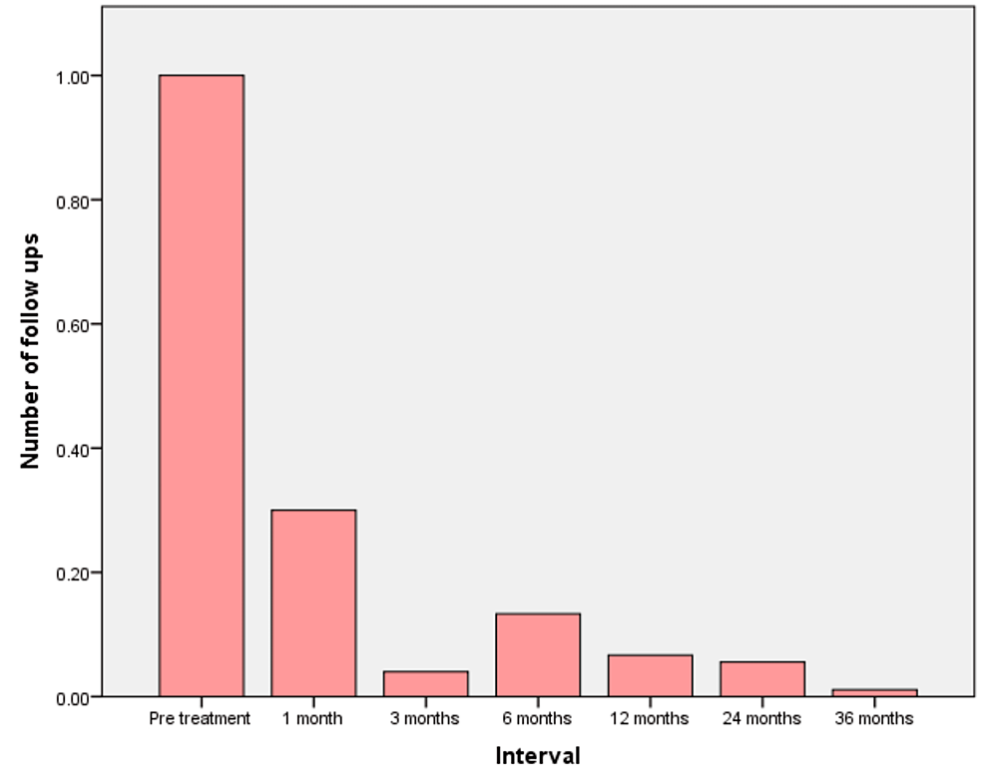
Graph representing the observed decrease in the number of follow-ups in the postoperative period

The preoperative quality-of-life assessment included 90 patients. Of these 90 patients, 41 (46%) have undergone surgery. Out of the 49 patients for whom surgery has not been done, only two have come for follow-up. The mean QoL was found to have no variation among these two patients. Sixteen out of 41 patients did not participate in the QoL assessment. Immediate postoperative QoL was assessed for 25/41 (60%). Twelve out of 25 cases (48%) have undergone follow-up after three months. Only six (24%), five (20%), and one (4%) patients have reported for follow-up after 12 months, 24 months, and 36 months, respectively, for review and assessment of QoL. A significant decrease in patients reporting for follow-ups after the treatment (p=0.00) was observed (Figure 2).

The mean QoL before the treatment was 4.64, and we witnessed a decline in QoL scores, with a mean score of 4.25 immediately after the treatment of OSCC. After one month, the mean QoL was 4.05. It was 4.91 after three months, 5.22 after six months, and 4.33 after 12 months. A gradual improvement in QoL scores was observed after 24 months, with a mean score of 5.8. After 36 months, the mean QoL score declined to 3. It was not statistically significant (p= 0.32) (Table 1).

**Table 1:** Showing the mean quality of life (QoL) observed in this study

Interval	Pre-treatment	Immediate postoperative	One month	3 months	6 months	12 months	24 months	36 months
Mean QoL	4.64	4.25	4.05	4.91	5.22	4.33	5.8	3

**Figure 3:**
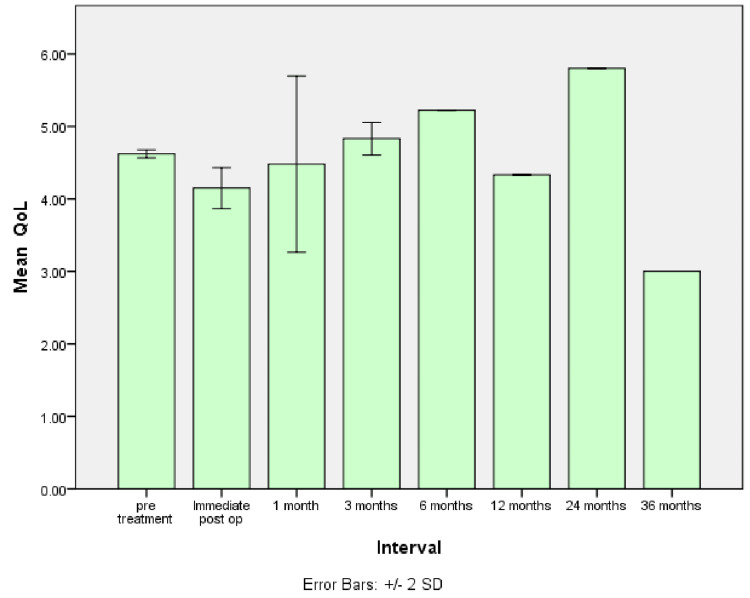
Graph shows the mean preoperative and postoperative QoL scores of OSCC patients obtained at different intervals QoL: quality of life; OSCC: oral squamous cell carcinoma

Figure 3 shows the mean preoperative and postoperative QoL scores of OSCC patients obtained at different intervals: immediate postoperative, one month, three months, six months, 12 months, 24 months, and 36 months.

**Figure 4:**
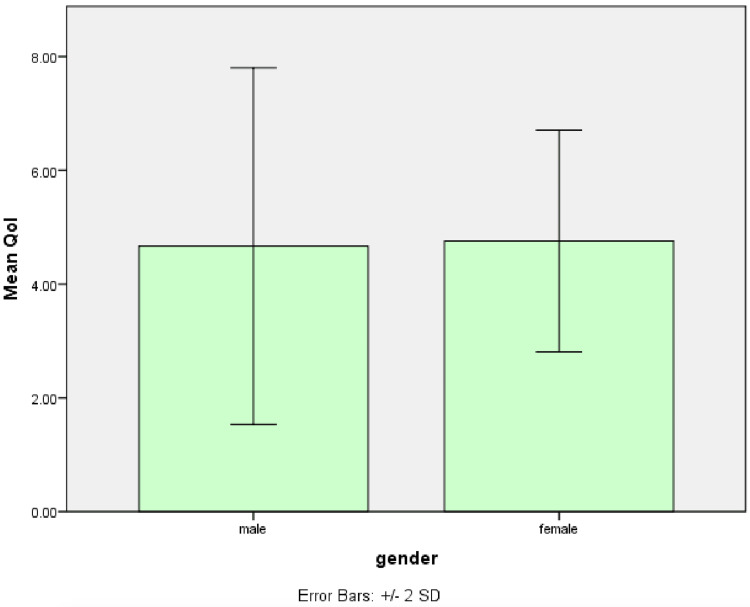
Graph shows the mean preoperative QoL score between different gender QoL: quality of life

The mean preoperative QoL in female patients was slightly higher, with a mean of 4.76, and in male patients, with a mean of 4.67. It was not statistically significant (p=0.157) (Figure 4).

**Figure 5:**
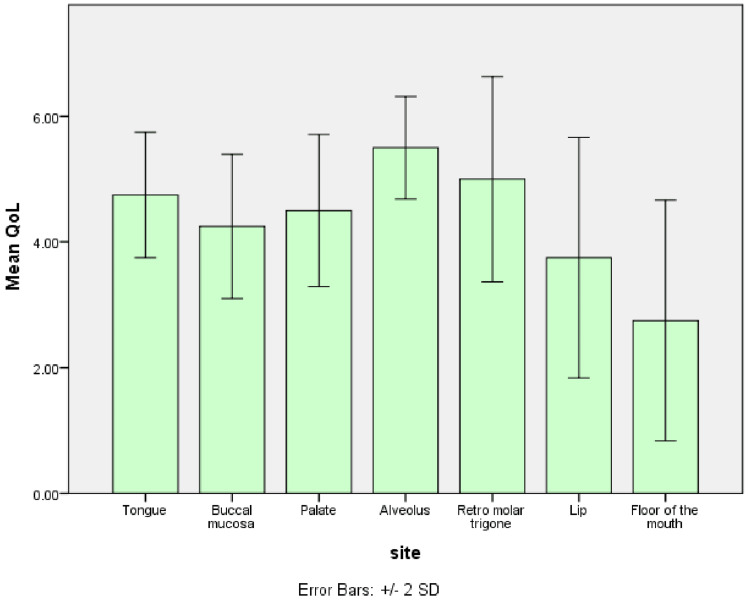
Graph shows the mean preoperative QoL score in OSCC patients of different sites QoL: quality of life; OSCC: oral squamous cell carcinoma

The mean preoperative QoL in patients with OSCC of the floor of the mouth was 3, followed by the lip (4), buccal mucosa (4.32), palate (4.33), tongue (4.89), retromolar trigone (5), and alveolus (5.33). It was not statistically significant (p=0.227) (Figure 5).

**Figure 6:**
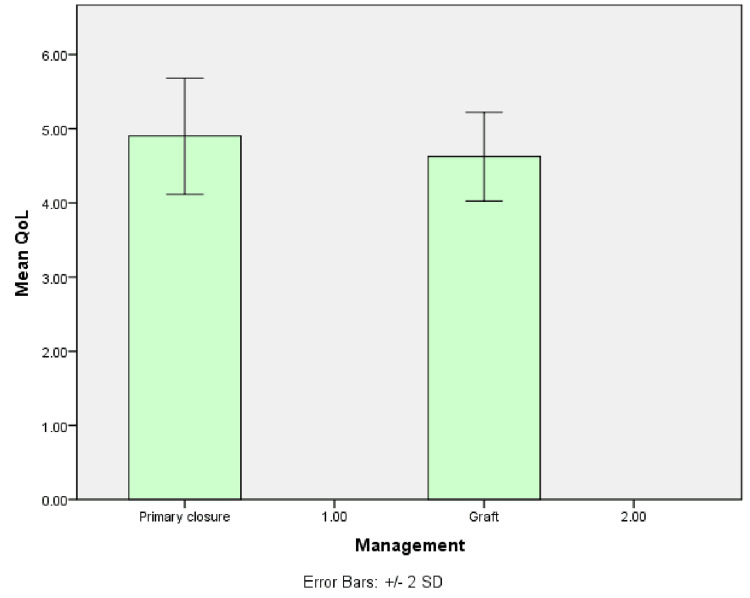
Graph shows the mean QoL score of patients who underwent only primary closure and those who underwent graft placement. QoL: quality of life

Patients who underwent only primary closure after excision had a mean post-op QoL score of 4.9, compared to those who underwent graft placement following excision, who had a mean score of 4.6. It was not statistically significant (p=0.157) (Figure 6).

**Figure 7:**
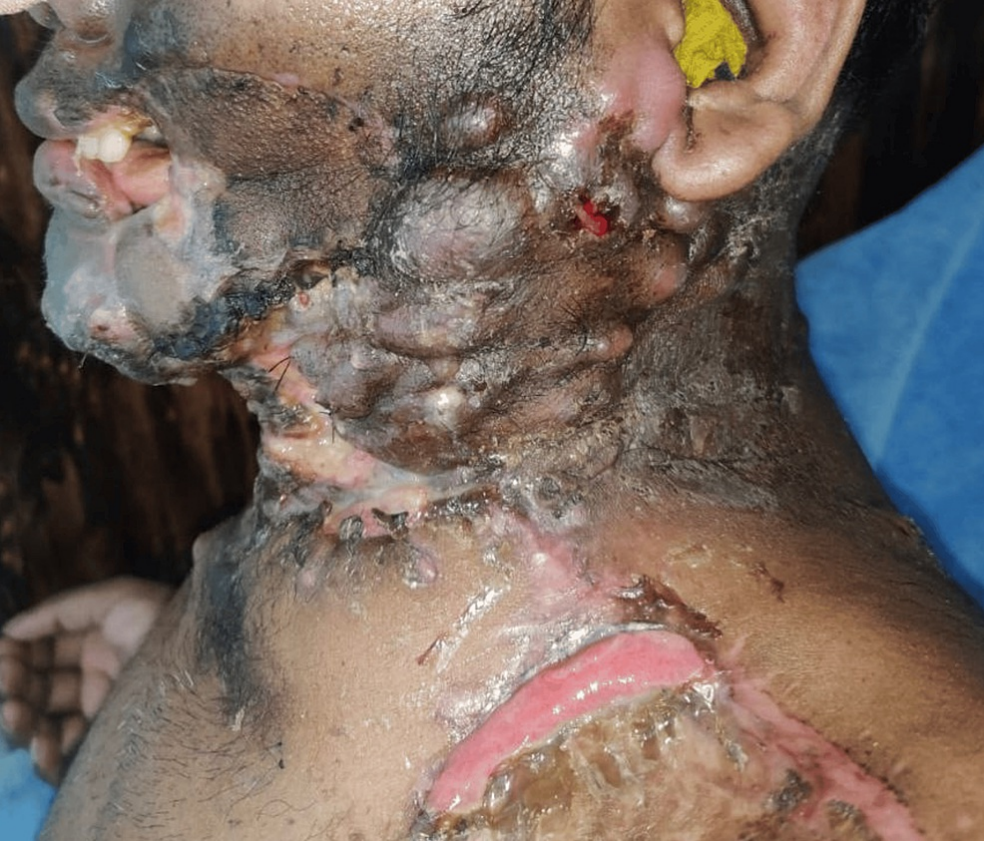
Clinical picture of a patient who underwent surgical excision with graft replacement and postoperative radiotherapy reported with secondary disease.

Out of the 41 patients, five (12%) succumbed to the disease. In four out of five patients (80%), the site of OSCC was buccal mucosa, and one patient had SCC of the tongue's lateral border (20%). Three of the patients (60%) also had skin involvement during surgery, and all the patients (100%) had T4a staging at the time of surgery (Figure 7).

## Discussion

We also observed that only 46% of our OSCC patients underwent surgery. We could attribute this to a lack of post-treatment support for such OSCC patients. The central government and state governments have issued medical insurance for cancer management. However, lack of awareness, regional beliefs, and fear of losing jobs could deter them from undertaking treatment.

Simon et al., in their study, found that in head and neck cancer patients, there is a higher follow-up frequency following the first two years, which subsequently decreases after five years [11]. These results are consistent with the findings of our study. The results of our investigation revealed a progressive decrease in the number of postoperative follow-ups among oral cancer patients. Treatment costs, reasons, caretaker issues, the distance from the hospital, and social stigma can all lead to indecision and improper follow-up.

Lack of follow-up makes it more difficult to create a proper strategy. Further, focused interventions for postoperative care and meticulous follow-up plans, especially for inoperable oral cancer patients, are also affected. There is a necessity for specialized approaches to reduce the lack of follow-up procedures, which calls for a serious reevaluation of the healthcare system's ability to enroll and keep oral cancer patients in long-term care plans [12].

The results also showed a decline in QoL immediately post-treatment, with a gradual improvement after two years. The initial QoL scores were low and could be due to the complex interplay of various factors during this critical period, such as physical symptoms and functional limitations, with psychological impact being the most significant cause. In a study by Blanco et al., a rise in the symptoms scale, which included pain, exhaustion, and weight loss, and a decrease in the functional scale, which included loss of role performance and physical, social, and emotional functions, were observed [13]. Scharloo et al., in prospective research with 177 patients, found that during the follow-up period, emotional function improved while social function deteriorated after oral cancer treatment [14]. In another study, the six-month post-treatment assessment revealed that 21.7% of patients had full or sub-syndromal post-traumatic stress disorder (PTSD) associated with cancer [15]. The subsequent recovery phase, characterized by a gradual improvement in QoL in our study, could also be influenced by factors like adaptation, rehabilitation, and psychological support, emphasizing the multifaceted nature of recovery in oral cancer survivors and contributing to this positive trajectory in their overall QoL.

In female OSCC patients, the mean preoperative quality of life was slightly higher than in male patients. This could be because women may have distinct coping strategies or social networks that enhance their quality of life while undergoing cancer treatment and recovery [16]. Furthermore, the patients with OSCC of the floor of the mouth had the lowest mean quality of life score, followed by buccal mucosa, palate, and lip. The site of the oral cancer may impact functions like breathing, speaking, swallowing, eating, and swallowing and may affect social interactions. The proximity of the tumors in the buccal mucosa and floor of the mouth to important structures may cause a significant disruption in their functions compared to other sites [17].

Patients who underwent only primary closure after excision had better QoL than those who underwent graft placement after the excision. This could be due to the faster recovery time and reduced complications after primary closure [18]. Compared to primary closure, grafting may result in visible scars with tissue texture or color changes. It can lead to a more unattractive cosmetic outcome [19]. There is an increased risk for donor site morbidity, problems in uptake at the graft site, the need for a second surgery for flap autonomization, and surgical defects until healing, which cause salivary contamination and may cause secondary infection, which may result in graft failure [20]. 

The lack of longitudinal follow-up studies reported in the Indian population highlights a significant gap in our understanding of the long-term impact of oral cancer and its impact on patients's quality of life (QoL) [21]. Standardized protocols should be established for longitudinal assessment to ensure comprehensive monitoring and evaluation of patients’s QoL over time [22]. Cultural, social, and psychological factors in developing countries like India should be noted. We emphasize the necessity for customized QoL questionnaires that have to be designed to capture the challenges faced by Indian patients.

Novel nanogels are being experimented with on oral cancer cell lines [23] and could potentially enter clinical trials soon. Artificial intelligence-based diagnosis of suspicious oral lesions [24] and estimation of QoL are also being explored [25]. Metronomic chemotherapy is also being experimented with as a viable treatment option for OSCC in India [26]. Additionally, there is a critical need for pre-and post-treatment counseling programs for oral cancer patients, along with their guardians, to provide holistic support and guidance throughout the treatment journey.

Establishing robust support systems is imperative to address the multifaceted needs of patients and enhance their overall well-being. Furthermore, efforts to raise awareness about government schemes and support mechanisms are essential to ensure access to healthcare resources and services. By addressing these key areas, we can strive towards improving the quality of life and outcomes for oral cancer patients in India.

The limitation of this study is the lack of postoperative patient follow-up for all enrolled patients, which may result in incomplete data representation. This could affect the precision of the quality of life evaluations conducted before and after surgery, potentially distorting the findings and understating the actual impact of the interventions. Thus, maintaining continuous patient follow-up is essential to understanding the long-term results.

## Conclusions

The longitudinal assessment of QoL in oral cancer patients revealed a decline in post-treatment QoL followed by a subsequent, gradual recovery. The mean Quality of Life Index consistently portrayed a lower state in the postoperative phase compared to the preoperative baseline, highlighting the enduring impact of oral cancer and its treatment on patients. The study concludes by emphasizing the need for ongoing research to explore specific interventions that can contribute to sustained improvement in QoL and personalized, holistic care approaches for this patient population. The multifaceted nature of QoL in oral cancer survivors calls for a continued commitment to research aimed at enhancing the well-being of individuals navigating the complexities of post-treatment recovery.
